# Supplementary material for: Exploring Psychological Factors for COVID-19 Vaccination Intention in Taiwan
Source: Vaccines (Basel). 2021 Jul 8;9(7):764. doi: 10.3390/vaccines9070764 (PMC8310074; doi:10.3390/vaccines9070764)
Supplement: Supplementary file 1 [file vaccines-09-00764-s001.zip › vaccines-1274946-supplementary.pdf]

The Survey Questions

Construct	Items
Mental models of virus	I think the coronavirus was artificially produced, and spread deliberately.
	I think the coronavirus was artificially produced, and spread accidentally.
	I think the coronavirus emerged naturally, and spread deliberately.
	I think the coronavirus emerged naturally, and spread accidentally.
Positive attitudes towards COVID-19 vaccines	A coronavirus vaccination should be mandatory for everyone who is able to have it.
	If I get a coronavirus vaccination, I will be protected against coronavirus.
	If I don’t get a coronavirus vaccination and end up getting coronavirus, I would regret not getting the vaccination.
	Other people like me will get a coronavirus vaccination.
	My family would approve of my having a coronavirus vaccination.
	My friends would approve of my having a coronavirus vaccination.
	A coronavirus vaccine will allow us to get back to ‘normal’.
Negative attitudes towards COVID-19 vaccines	I would be worried about experiencing side effects from a coronavirus vaccination.
	I might regret getting a coronavirus vaccination if I later experienced side effects from the vaccination.
	A coronavirus vaccination will be too new for me to be confident about getting vaccinated.
Belief of vaccine-induced infection	A coronavirus vaccination could give me coronavirus.
Powerlessness	I feel that the coronavirus is too big for my actions to have an impact.
	I feel that my actions will not affect the outcome of coronavirus.
	I feel that my contribution is just a drop in the ocean and so is insignificant.
Vaccination intention (General)	If COVID-19 vaccines are available in Taiwan, I will get vaccinated.
Vaccination intention (Source specific)	If social media promote COVID-19 vaccines, I will get vaccinated.
	If friends around me encourage me to get a COVID-19 vaccine, I will get vaccinated.
	If the government promotes COVID-19 vaccines, I will get vaccinated.
	If health professionals (doctors or nurses) promote COVID-19 vaccines, I will get vaccinated.
	If mainstream media promote COVID-19 vaccines, I will get vaccinated.
Demographic information	What is your gender?<br>*-“female”, “male”*
	What is your age?<br>*-“20-24”, “25-29”, “30-34”, “35-39”, “40-44”, “45-49”, “50-54”, “55-59”, and “60 and above”*
	Where is your city/county of residence?<br>*-22 local administrative divisions of Taiwan, and 1 option of “others”*
	What is your level of education?<br>*-“primary school”, “junior high school”, “senior high school”, “vocational school”, “bachelor”, “master or doctor”*
	Your monthly income?<br>*-“<20,000 NTD”, “20,000-40,000 NTD”, “40,000-60,000 NTD”, “60,000-80,000 NTD”, “80,000-100,000 NTD”, “100,000-120,000 NTD”, “120,000-140,000 NTD”, “140,000-160,000 NTD”, “160,000-180,000 NTD”, “180,000-200,000 NTD”, “>200,000NTD”, “I don’t know.”*
	What is your occupation?<br>*-“student”, “homemaker”, “retiree”, “agricultural, forestry and fishery”, “food service”, “manufacturing”, “health care”, “construction”, ”self employed”, “telecommunication service”, “financial and insurance service”, “public servant”, ”mass communication”, “information technology”, “hospitality”*
